# Notes and News

**Published:** 1903-02-07

**Authors:** 


### Notes and News.

Professor Virchow's successor as President of the Berlin Medical Society is Professor von Bergman.

Lord Roberts will be admitted as Honorary Fellow of the Royal College of Surgeons at the Hunter Festival Dinner on February 14th.

The Local Government Board are to be approached to urge the introduction of a Bill in Parliament providing for the efficient supervision and inspection of oyster beds. Dr. Collingridge, the Medical Officer of Health for the City, states that all the Thames shell fishery is under grave suspicion.

In the February number of *The World's Work*—which by the bye is a very interesting publication—there is a well-illustrated article descriptive of the extent to which the most advanced applications of science are employed in British hospitals for the cure of disease. Needless to say that the light treatment, electric baths, *x*-rays and operating rooms figure largely among the illustrations. We think that the writer is in error in stating that the *plenum* system of ventilation prevails throughout University College Hospital.

Sir Henry Burdett delivered one of his present series of addresses on behalf of the League of Mercy at the Paddington Baths on January 29th. The audience was one of the largest which has ever assembled in Paddington, where popular enthusiasm does not run very high. The meeting pledged itself to further the work of the hospitals through the League of Mercy. The following fixtures have been made by Sir Henry Burdett, the hon. treasurer, for future addresses:—The Town Hall, Hammersmith, Monday, February 9th, at 8 o'clock; the Town Hall, Lewisham, Thursday, February 12th, at 8 o'clock; the Royal Victoria Hall, Waterloo Bridge Road, Wednesday, February 18th, at 8 o'clock; the Memorial Buildings, Roscoe Street, Bunhill Row, Thursday, February 19th, at 8.30; the Whitechapel Art Gallery, High Street, Whitechapel, Tuesday, February 24th, at 8 o'clock.

Geneva is suffering under an epidemic of influenza, which is very universal but of a mild type.

An isolation hospital has been arranged for Rathmines and Pembroke in Ireland. The old hospital, which is close at hand, will serve as a residence for the staff.

King's College Hospital will probably receive £60,000 through the will of the late Mr. Robert R. Storks, of Twickenham. This munificent legacy will come in very opportunely if the hospital carries out the scheme of removing to Camberwell or elsewhere.

At a meeting held at Berlin last Saturday in connection with the forthcoming Fire Exhibition in London, at which Germany is to be represented, the Colney Hatch fire was referred to. The opinion was expressed that England does not devote enough attention to the subject of fire prevention, and that regulations in Germany would have rendered such a disaster impossible.

The Royal Eye Hospital has made complaints as to the treatment it has received at the hands of the Lambeth Waterworks Company in cutting off the water supply of the hospital. The Company, however, explain that the supply has not been cut off by them, but that it must have been tampered with. They offer to co-operate in the prosecution of anyone found interfering unlawfully with the water supply of the hospital.

The Hospital Saturday Fund are distributing £20,252 this year amongst the medical institutions. They give £7,400 to general hospitals, £283 to cottage hospitals, £6,180 to special hospitals, £971 to dispensaries, £1,838 to convalescent homes, and £3,576 to miscellaneous institutions. They have made the largest award this year to the London Hospital, amounting to £1,697; next on the list comes St. Mary's with £540.

An important decision affecting public vaccinators all over the country, was given by Mr. Sidney Jerrold, Local Government Board Auditor, at Kingston-on-Thames on Thursday on the subject of the charges by public vaccinators of the union for revaccinations. It appeared that between May 28th and June 6th some 493 men were revaccinated at the Chelsea, Lambeth, and other waterworks at Surbiton, and the charge made for each was 7s. 6d. This, it was contended, was illegal, inasmuch as the vaccination did not take place at the patients' homes. The auditor said the lower fee of 2s. 6d. per head was payable when vaccination was performed at the surgery, or "elsewhere" than at the home of the person vaccinated, and he came to the conclusion that the fees paid for vaccination performed at any place other than at the home of the person vaccinated must be paid for on the lower and not the higher scale. The question was whether vaccination was performed at home or not. It was scarcely arguable that when a large number of people were vaccinated at a factory, or in works in or out of doors, they could be said to be at home. He should, therefore, disallow the difference between the lower and higher scale—2s. 6d. and 7s. 6d.

